# Prevalence of anemia and related factors among women in Turkey

**DOI:** 10.12669/pjms.332.11771

**Published:** 2017

**Authors:** Birsen Karaca Saydam, Rabia Ekti Genc, Fulden Sarac, Esin Ceber Turfan

**Affiliations:** 1 Dr. Birsen Karaca Saydam, BKS. Ege University Faculty of Health Sciences, 35100 Bornova, Izmir, Turkey; 2 Dr. Rabia Ekti Genc, REG. Ege University Faculty of Health Sciences, 35100 Bornova, Izmir, Turkey; 3 Prof. Dr. Fulden Sarac, FS. Department of Internal Medicine, Ege University Medical School, 35100 Bornova, Izmir, Turkey; 4 Prof. Dr. Esin Ceber Turfan, ECT. Ege University Faculty of Health Sciences, 35100 Bornova, Izmir, Turkey

**Keywords:** Anemia, Reproductive age, Women, BMI: Body Mass Index, TDHS: Turkey Demographic and Health Survey, WHO: World Health Organization

## Abstract

**Objective:**

To determine the prevalence of anemia and related factors among women in Turkey.

**Methods:**

This descriptive study was conducted at the outpatient clinics of the Department of Internal Medicine, Ege University Medical School. Randomly selected women were given questionnaires regarding their socio-demographic and obstetric characteristics. The data were coded and analyzed using SPSS version 17.0 software. Statistical analyses with 95% confidence intervals were considered to be significant if p<0.05.

**Results:**

The study results showed an anemia prevalence of 27.8% in the study sample. Among all anemia diagnoses among the participants, 56.0% were determined to have iron deficiency, 37.1% iron-deficiency anemia, and 6.9% severe anemia. It was observed that anemia was detected among women who were 15-49 years of age (p<0.05), menstruating (p<0.05), had a history of Cesarean section (p<0.05), and had not entered menopause (p<0.05). Based on forward-stepwise-logistic regression analysis, the most important parameter was concluded to be age group, which was followed by menopausal status.

**Conclusions:**

The study results suggest that the anemia prevalence rate is specifically higher among women of reproductive age. To prevent anemia at a low cost, it is recommended to provide women with relevant information and well-planned interactive educational programs.

## INTRODUCTION

Anemia is a condition in which levels of hemoglobin, hematocrit, and erythrocytes fall below the normal range. The World Health Organization (WHO) defines anemia as hemoglobin concentrations below 12 g/dL in women and 13 g/dL in men.^1^,^2^ The prevalence of anemia increases during growth and development when there is an increased need for an iron-rich diet.^3^,^4^ More than 30% of patients admitted to hospitals in developed countries are reported to be anemic, and this rate is known to be higher in developing countries and among women.^4^

Anemia is more prevalent among women than men, and according to the WHO, the anemia prevalence among women is 21-80% worldwide.^4^,^5^ Anemia, which already negatively impacts life, is also an important factor that negatively impacts the health of women and their ability to work, particularly in their reproductive years, and leads to increased infant and maternal mortality.^3^,^6^,^7^ According to the WHO, 40-89% of anemia among women is characterized as iron-deficiency anemia. This rate is 22.9% in Europe and 24.3% in Turkey.^2^

Iron-deficiency anemia is observed when dietary intake of iron declines, when iron is not sufficiently absorbed, when bodily requirements increase, or in cases of excessive blood loss.^8^,^9^ Economic analyses show that iron-deficiency anemia can be easily cured with low-cost measures, such as through the provision of dietary education at diagnosis. Severe anemia develops when iron deficiency is not prevented and/or controlled, which impacts a woman’s physical, mental, and social health and becomes a significant public health issue.^7^,^10^,^11^ Any visit a woman makes to the hospital for any type of complaint, especially when blood samples are drawn for diagnostic purposes, is an opportunity to evaluate the patient with respect to anemia and to provide them with need-based treatment, dietary and lifestyle advice, and reproductive health information and precautions. Any study conducted on this subject is of significance in terms of public health and enhancing women’s health.

The aim of this study was to determine the prevalence of anemia and related factors among women in Turkey.

## METHODS

This descriptive cross-sectional study was conducted in the Internal Disease Department of Ege University School of Medicine. Ege University Hospital is located in Izmir, the 3rd largest city in Turkey, and serves patients from all socioeconomic classes. The Institutional Review Board of the Ege University School of Health Sciences and the Head of the Internal Disease Department of Ege University Medical School approved the study protocol.

The sample size was calculated using the n=t^2^pq/d^2^ formula for unknown population sizes, taking 24.3% as the prevalence for anemia among women^2^ [*n: sample, p: the prevalence for anemia among women in Turkey, q: women without anemia in Turkey (1-p), t: theoretical t value, d: standard error*]. The formula provided a minimum sample size of 282.66 [t=1.96, p=24.3%, q=75.7% (1-24.3), d=0.05]. The target sample size was set at 283. All women (n=432) who visited the outpatient clinic between April 20 and June 20, 2016, were interviewed. The women were informed about the aim of the study, and those who were over 15 years of age, were not gestational, had visited the clinic for the first time, and consented to participate (n=418) were included in the study (Participation rate: 96.7%). All participants signed an informed consent form.

### Definition of anemia

Blood samples were drawn by the central laboratory staff according to standard operating procedures upon the request of a physician. The blood samples were analyzed at the Biochemistry Department, Ege University Medical School. The hemogram (hemoglobin, hematocrit, iron, and iron-binding capacity) findings were included in the analyses in this study. An independent hematologist evaluated the results to diagnose anemia. Hemoglobin levels below 12 g/dL were considered as anemia according to the WHO anemia classification for non-gestational adult women.^2^

“Iron deficiency” was defined as having blood iron levels below the normal range without anemia (normal hemoglobin values). “Iron-deficiency anemia” was defined as having both low blood iron levels and anemia (hemoglobin values below normal limits). “Severe anemia” was defined as having low blood iron levels and hemoglobin ≤7 g/dL.

### Statistical analysis

Collected data were coded and analyzed with SPSS version 17.0 software. Chi-squared (χ^2^) and Fisher’s exact tests were used for the comparison of categorical data, while the Kruskal Wallis test, the Mann Whitney U test, and Logistic Regression were used in analysis of numerical data. Logistic regression analysis was conducted to identify explanatory variables. Data were expressed as “mean (standard deviation; SD)” and percent (%) where appropriate. p<0.05 was considered statistically significant.

## RESULTS

Overall anemia prevalence among the study subjects was determined to be 27.8%. It was observed that out of all anemia diagnoses among these women, 56.0% were iron deficiency, 37.1% were iron-deficiency anemia, and 6.9% were severe anemia; these rates for all women were 15.6%, 10.3%, and 1.9%, respectively. The mean age of the women was 45.16±16.21 years (median=45.0; min=18.0, max=89.0).

Among all participants, 56.5% were in the 15-49 years age group, 34.7% were elementary school graduates, 80.6% were unemployed, 65.8% were married women, 62.0% had low incomes compared to expenses, 81.1% were living with a nuclear family, 86.1% were living with non-relative parents, and 75.4% had lived in a town for the majority of their lives (Table-I).

**Table-I:** Distribution of participants by sociodemographic characteristics.

*Sociodemographic characteristics*	*n*	*%*
***Age group***		
15-49 years	236	56.5
≥50 years	182	43.5
***Level of education***		
Literate	37	8.8
Elementary school	145	34.7
Middle school or equivalent	47	11.2
High school or equivalent	113	27.1
University or higher	76	18.2
***Employment***		
Employed	81	19.4
Unemployed	337	80.6
***Marital status***		
Single	74	17.7
Married	275	65.8
Divorced or widowed	69	16.5
***Income level***		
Income < expenses	259	62.0
Income = expenses	152	36.4
Income > expenses	7	1.6
***Family type***		
Nuclear	339	81.1
Extended	24	5.7
Separated	55	13.2
***Parents’ kinship***		
Relative	58	13.9
Non-relative	360	86.1
***The longest place of residence***		
Village	17	4.0
Town	315	75.4
Province	86	20.6

Total	418	100.0

According to their obstetric characteristics, 53.4% were menstruating women, 75.4% were women with a history of pregnancy, 46.0% had no miscarriage or abortion history, 70.3% had given birth, 55.5% had a history of vaginal birth, 31.8% had 2 living children, and 54.8% were premenopausal women (Table-II).

**Table-II:** Distribution of participants by obstetric characteristics.

*Obstetric characteristics*	*n*	*%*
***Menstruation status***		
Menstruating	223	53.4
Not menstruating	195	46.6
***Pregnancy***		
Pregnant	315	75.4
Non-pregnant	103	24.6
***Miscarriage / abortion history***		
Yes	192	46.0
No	226	54.0
***History of delivery***		
Given birth	294	70.3
Have not given birth	20	4.8
Sub Total	314	75.1
***Delivery Method***		
Vaginal	232	55.5
Cesarean section	62	14.8
Sub Total	294	70.3
***Number of living children***		
1	57	13.6
2	133	31.8
3	44	10.5
4 or more	50	12.0
Sub Total	284	67.9
***Menopausal status***		
Post-menopausal	189	45.2
Pre-menopausal	229	54.8

Total	418	100.0

For all women, the mean time between pregnancies was 3.02±2.27 years (n=418; median=2; min=1.0, max=14.0), the average age at first delivery was 22.41±4.97 years (n=294; median=21; min=14.0, max=40.0), the average number of births was 2.59±1.50 (n=294; median=2; min=1.0, max=9.0), and the average number of living children was 2.42±1.26 (n=284; median=2; min=1.0, max=8.0).

The relationship between BMI and anemia was also evaluated. It was observed that 27.6% of the women in the group with a BMI ≥24.5 kg/m^2^ were anemic. The difference between groups was not statistically significant (χ^2^=0.571, df=2, p=0.770).

With respect to their habits, 19.8% women were smokers, 24.1% women consumed alcohol, 22.7% were using oral contraceptives, 28.5% were using intra uterin devices, and 28.4% of the women who reported a regular eating pattern received an anemia diagnosis.

Upon further detailed analysis of eating habits, it was observed that among women with anemia, 31.5% consumed vegetables once per week or less, 38.2% consumed red meat very rarely, 38.8% consumed white meat very rarely, 30.4% consumed fruits very rarely, 42.9% consumed carbohydrates very rarely, 34.8% consumed legumes once every two weeks, 33.4% consumed milk and dairy products once a week, and 29.4% consumed tea/coffee a few times a day.

The factors impacting anemia diagnosis among the women included in the study were compared based on the type of anemia. Among the anemic women in the 15-49 year age group, 16.5% were diagnosed with iron deficiency, 15.3% with iron-deficiency anemia, and 3% with severe anemia (χ^2^=20.137, df=3, p=0.000); among the menstruating anemic women, 15.2% were diagnosed with iron deficiency, 14.8% with iron-deficiency anemia, and 3.1% with severe anemia (χ^2^=15.186, df=3, p=0.002); among those who had given birth via Cesarean section, 19% were diagnosed with iron deficiency, 28.6% with iron-deficiency anemia, and 9.5% with severe anemia (χ^2^=27.583, df=6, p=0.000); and among the postmenopausal participants, 14.8% were diagnosed with iron deficiency, 4.2% with iron-deficiency anemia, and 0.5% with severe anemia (χ^2^=19.160, df=3, p=0.000) (Table-III).

**Table-III:** Comparison of factors impacting anemia classification.

*Characteristics of anemic women*	*Iron deficiency %*	*Iron deficiency anemia %*	*Severe anemia s%*	*χ^2^*	*df*	*p*
Age 15-49 years	16.5	15.3	3.0	20.137	3	0.000
Menstruating	15.2	14.8	3.1	15.186	3	0.002
History of Cesarean section	19.0	28.6	9.5	27.583	6	0.000
Non-menopausal	14.8	4.2	0.5	19.060	3	0.000

The study findings showed that anemia was associated with an age younger than 50 years, menstruation, delivery via Cesarean section, and being in a premenopausal state. These risk factors were investigated by multivariate logistic regression, and the results are presented in Table-IV. According to the logistic regression analysis, being younger than 50 years of age (15-49 years) increases the risk of anemia by 2.7 times [odds ratio=2.727, 95% confidence interval (Expβ)=0.559-11.584, p=0.000], and not being in the premenopausal state increases the risk by 2.4 times [odds ratio=2.486, 95% confidence interval (Expβ)=1.7806-18.3113, p=0.001]. Logistic regression analyses also determined that menstruation and history of Cesarean section may also increase the risk of anemia; however, this increase was not found to be statistically significant in this study (Table-IV).

**Table-IV:** Distribution of influence of anemia-affecting factors.

*Variable*	*Logitβ*	*SE*	*Wald*	*df*	*OR*	*95%CI Expβ*	*p*
Age 15-49 years	1.003	0.271	13.722	1	2.727	1.604-4.637	0.000
Pre-menopausal	0.910	0.270	11.374	1	2.486	0.559-11.584	0.001
History of Cesarean section	0.575	0.338	2.897	1	1.777	0.917-3.447	0.890
Menstruating	0.934	0.773	1.461	1	2.546	1.464-4.219	0.227

## DISCUSSION

The study findings indicate a prevalence of 27.8% for anemia. Prevalence of iron-deficiency anemia, on the other hand, is reported as 10.3%. Among the women diagnosed with anemia, 19.6% were postmenopausal, and 34.4% were premenopausal. According to the WHO’s anemia prevalence figures for 1993-2005, 30.2% of non-pregnant and 41.8% of pregnant women are anemic.^5^ Bodnar et al.’s study of women in low-socioeconomic conditions during the postpartum period reported an anemia prevalence of 27.0%.^12^

Anemia is widespread in developing countries in Asia, Africa, South America, and even in Western Europe. A systematic review of studies conducted between 1995 and 2011 shows a 4.0% decline in cases of anemia globally.^13^ The same study also reported a decline from 33.0% to 29.0% among non-pregnant women and a decline from 43.0% to 30.8% among pregnant women. These rates of decline are promising but not suffcient. The fight against anemia calls for detection of the issue first and identification of risk factors in different countries and regions. Additionally, due to great improvements in different countries based on their social, economic and cultural differences, the rate of anemia has been decreasing compared to previous years.^14^-^16^

The prevalence of iron-deficiency anemia caused by eating disorders, infectious diseases, impairments in iron intake or absorption, or factors associated with the gastrointestinal system can vary even in developed countries between 11-30% across patient groups with different socio-demographic characteristics.^7^,^17^ According to the National Food and Nutrition Strategy Report of 2003 by the Prime Ministry State Planning Organization of the Republic of Turkey, iron deficiency is the leading (90.0%) cause of anemia in Turkey.^18^ The Health Statistics 2013 Report by the Ministry of Health indicates that 9.8% of Turkish women reported that they have experienced iron-deficiency anemia.^19^ The reasons for a higher prevalence of iron-deficiency anemia among women are menstrual irregularities and a high number of frequent births.^20^

The prevalence of iron-deficiency anemia in our study was found to be 37.1%. Steven et al.’s systematic review on the effects of diet on severe anemia among pregnant and non-pregnant women reports a prevalence of anemia of 29.0%.^13^ Iron-deficiency anemia was reported to be the cause of 12.8% of maternal mortality, especially in Asia. This fact underlines that iron-deficiency anemia with a high prevalence (37.1%) constitutes a vital risk for Turkish women of reproductive age.^21^

Studies have shown higher anemia prevalence among women with a BMI at obesity levels.^22^ According to our findings, 27.6% of the women with an anemia diagnosis were obese. Interestingly, 72.4% of the women without anemia were also obese. This can be explained by severe malnutrition and increased weight gain following menopause. Kara et al. detected a slightly significant negative correlation between BMI and serum iron levels (r=-0.234, p=0.027) among obese women of reproductive age.^23^ In contrast to their findings, despite the high rates observed in our study, statistical analyses showed that the difference was not statistically significant (χ^2^=0.571, df=2, p=0.773). Socioeconomic factors are reported to be closely associated with anemia.^24^ Martinez et al. completed a study on the effects of socioeconomic factors influencing anemia development aiming to lower the prevalence in Afghanistan. In terms of wealth distribution, in the aforementioned study, they reported that women who survive through agriculture and animal breeding have a lower prevalence of anemia.^25^ Our study did not determine a statistically significant difference in the prevalence of anemia based on income level. However, an anemia prevalence of 38.2% among women who consume red meat very rarely supports the findings of Martinez et al., albeit indirectly.^25^

In our study, more than one out of four (28.5%) women who use cervical contraceptive tools (Copper T 380A) were anemic. Studies globally recommend anemic women use contraceptive methods that reduce bleeding in regard to safe maternity decisions and prevention.^26^,^27^

Anemia among women of reproductive age is among the more significant causes of maternal mortality and is associated with number, frequency and method of deliveries. In our study, approximately one out of three women who had 3-4 or more children were anemic, 29.5% and 28%, respectively. The statistical analyses between anemic and non-anemic women, delivery method and menopause status yielded a significant difference. Further analyses indicated this difference stems from Cesarean section births. Cesarean sections have been steadily increasing (multiplying each year) over the past 10 years in Turkey. According to Turkish Public Health data, Cesarean section deliveries constitute 50.0% of births. Since this rate, is 24.0% for the WHO European region, is of great concern.^19^ Our study also showed that elective Cesarean section deliveries, not associated with any medical indication, are highly associated with the development of anemia, in addition to the various medical and economic complications they are also associated with.

### Limitations of the study

Lack of follow-up data on hemogram findings of patients who were diagnosed with anemia and then given education/counselling by researchers seems to be an important limitation that otherwise would extend the knowledge achieved in the current study.

## CONCLUSION

The prevalence of anemia was found to be 27.8%, and the risk for anemia was increased among women between 15-49 years of age (2.7 times higher), menstruating (and premenopausal) (2.4 times higher), and with a history of Cesarean section. These findings suggest a causal relationship between the reproductive process and the risk of anemia development. The women diagnosed with anemia were provided educational support and were informed about anemia treatment and risk factors.

### Authors’ Contributions

**BKS and REG** contributed to the conception/design of the research.

**BKS** acquisition of the data and literature research.

**REG and FS** analysis and interpretation of the data.

**BKS** drafted the manuscript.

**BKS, FS and ECT** critically revised the manuscript.

**FS and ECT** provided intellectual content of critical importance to the work.

**BKS** had primary responsibility for final content. All authors read and approved the final manuscript.
